# Cost‐effectiveness of the diabetes remission clinical trial (DiRECT)/Counterweight‐Plus weight management programme, based on 5‐year follow‐up

**DOI:** 10.1111/dme.70306

**Published:** 2026-03-29

**Authors:** A. Davies, E. Grieve, L. McCombie, A. McIntosh, A. McConnachie, W. S. Leslie, N. Sattar, R. Taylor, M. E. J. Lean

**Affiliations:** ^1^ Health Economics and Health Technology Assessment, School of Health and Wellbeing, College of Medical, Veterinary & Life Sciences University of Glasgow Glasgow UK; ^2^ Human Nutrition, School of Medicine, Dentistry & Nursing, College of Medical, Veterinary & Life Sciences University of Glasgow Glasgow UK; ^3^ Robertson Centre for Biostatistics, School of Health and Wellbeing, College of Medical, Veterinary & Life Sciences University of Glasgow Glasgow UK; ^4^ School of Cardiovascular and Metabolic Health, College of Medical, Veterinary & Life Sciences University of Glasgow Glasgow UK; ^5^ Newcastle Magnetic Resonance Centre, Institute of Cellular Medicine Newcastle University Newcastle upon Tyne UK

**Keywords:** diabetes, economics, type 2 diabetes

## Abstract

**Aims:**

Follow‐up of the Counterweight‐Plus intervention in DiRECT has demonstrated 6.1 kg weight loss and 10 % remissions from type 2 diabetes at 5 years. We previously reported cost‐effectiveness based on 2‐year results. We now present an updated cost‐effectiveness analysis, based on available 5‐year data.

**Methods:**

A lifetime cost‐effectiveness analysis was conducted using the 5‐year data from DiRECT, including time to relapse from remission from type 2 diabetes, intervention costs (practitioner appointment visits, low‐energy formula diet sachets), routine healthcare resource use (primary, secondary, and tertiary care contacts), medications (anti‐hypertensive and anti‐diabetic medicines), and quality of life (EuroQol EQ‐5D‐3L). Modelling assumed that all those in remission at 5 years would relapse after a maximum of 10 years.

**Results:**

Total (discounted) healthcare cost‐savings (excluding intervention costs) per participant over 5 years amounted to £2091. On average, healthcare costs per annum were ~£480 per participant lower in the intervention arm (*p* < 0.05). Intervention resource use beyond 2 years added relatively little to the total intervention cost (set‐up, formula diet and practice visits), which amounted to £1691 (95% CI: £1566, 1822) per participant over 5 years. Reduced hospital admissions accounted for the major part of this saving, with the remainder being across primary and secondary care, and anti‐diabetic and anti‐hypertensive medications. Remission in DiRECT was associated with a significant improvement in quality of life, and the intervention arm was modelled to achieve both cost‐savings of ~£400 and quality adjusted life year gains of 0.043 over 5 years, with further benefits due to reduced mortality in remission. Over the full lifetime horizon of the analysis the intervention was modelled to produce 0.08 incremental QALYs, saving £496 per participant.

**Conclusions:**

The costs involved in achieving remissions from type 2 diabetes with the Counterweight‐Plus diet programme were offset within 5 years through reduced healthcare resource use. Though remission may ultimately relapse, sustained weight loss brings other longer term health gains. The intervention, even using one‐to‐one face‐to‐face contacts rather than remote delivery, which is increasingly adopted, generated both health gains and cost‐savings compared with current best practice.

**What's new?**

**What is already known?**

- Economic analysis at 2 years predicted the DiRECT intervention would dominate usual care that is, be both more effective, and less costly.

**What this study adds?**

- Although continued low‐intensity dietary support incurred marginally increased intervention resource use, this analysis extends the economic evidence on the reduced healthcare resource use and cost‐effectiveness associated with the benefits of more sustained intentional weight loss and remission of type 2 diabetes.

**What are the implications of the study?**

- Using longer term data on maintenance of remission and healthcare costs, this extension study greatly reinforces evidence on the cost‐effectiveness of the DiRECT intervention and the value for money of extending measures for routine management of type 2 diabetes through weight loss.

## 1 INTRODUCTION

In the Diabetes Remission Clinical Trial (DiRECT), delivery of a low‐energy diet‐based intervention in primary care for people with diabetes duration of less than 6 years achieved remission from type 2 diabetes in 46% and 36% of participants after 12 and 24 months, respectively.^1^, ^2^ Along with other studies,^3^, ^4^, ^5^ DiRECT has resulted in type 2 diabetes now being recognised as a reversible condition for most people. A recent systematic review and meta‐analysis has described the relationship between the degree of weight loss attained and the probability of remission from type 2 diabetes,^6^ reinforcing the findings in DiRECT.^7^ The Counterweight‐Plus programme was initiated with ‘total diet replacement’ using a low‐energy formula diet (soups and shakes) providing 825–853 kcal per day for 12–20 weeks (Counterweight, UK). This was followed by structured food reintroduction for 2–8 weeks and a subsequent longer term programme of weight loss maintenance. For relapse management, 2–4 week ‘rescue packages’ of the formula diet were offered if weight was regained or if diabetes returned. All oral glucose‐lowering and anti‐hypertensive medications were suspended on safety grounds at the start of the programme, and reintroduced if necessary using protocols based on blood glucose and blood pressure monitoring. Reversal of diabetes in DiRECT was strongly associated with weight loss. Over 70% of those achieving weight loss of 10 kg or more were in remission from diabetes at both 12 and 24 months.

Beyond 2 years, in an extension study, routinely collected UK National Health Service (NHS) data were captured for both the original control group and intervention groups, including those who had withdrawn from the study, providing longer term data on maintenance of remission and healthcare costs. Extended support (3‐monthly nurse visits) was offered to intervention participants for up to 5 years.

In the original control group under routine management, only 11 individuals (7%) achieved remission at any annual study assessment, and five (5%) of 93 were in remission at year 5.^8^ Among intervention participants, 73 achieved remission at any annual study assessment, and of participants with available data, 53 (41%) and 12 (10%) were in remission at 2 and 5 years, respectively. All participants, including the control group, were encouraged to consider weight management after results of the 2‐year study were reported. Thus, as previously noted, their outcomes may not reflect usual care.^8^ The intervention‐extension group was self‐selected, albeit by almost all those who were eligible, and the group differences reported for DiRECT were not interpreted as treatment effects. Nevertheless, the longer term follow‐up data provide valuable information with which to estimate the durability of greater remission demonstrated during the earlier randomised phase of DiRECT.

We previously reported a decision analytic model extrapolating long‐term costs and outcomes from the 2‐year results of DiRECT^9^ to assess whether the Counterweight‐Plus programme could be a cost‐effective intervention for people with type 2 diabetes. We projected durations of remissions, assuming continued relapse rates as seen in year 2 of DiRECT. We modelled gains in life expectancy and quality adjusted life years due to reduced mortality during periods of remission relative to type 2 diabetes. The analysis did not address the wider benefits beyond diabetes remission that might be derived from improved weight management, nor did it model the detailed clinical course of type 2 diabetes. Though these wider benefits were not considered, that analysis nonetheless predicted the DiRECT intervention would dominate usual care (i.e., be both more effective, and less costly), as the costs associated with delivering the Counterweight Plus intervention within routine care were more than offset by projected savings in healthcare resource use. These savings were estimated in part based on 2‐year data collected in DiRECT, with longer term healthcare costs based on published estimates. A notable result of the analysis was the intervention was projected not only to be cost‐saving over the long‐term, but to be cost‐neutral within 5 years.

Continued low‐energy diet support over the longer term will result in further intervention resource use beyond the randomised period of DiRECT for a proportion of people. Here, we present an updated cost‐effectiveness analysis based on maintenance of remission in DiRECT to 5 years, replacing our original intervention and healthcare cost and quality‐of‐life assumptions with estimates based on actual healthcare data and participants' EuroQol EQ‐5D data collected over the course of the study.

## 2 METHODS

We updated the modelling approach adopted for the 2‐year cost‐effectiveness analysis of DiRECT. The analysis aims to assess the cost‐effectiveness of the Counterweight Plus intervention, as evaluated in the population in DiRECT (diagnosis of diabetes within 6 years and with BMI over 25–27 kg/m^2^), as an alternative to routine care in achieving remission from type 2 diabetes. The model adopts a simple three state Markov structure, with states of type 2 diabetes, remission from diabetes, and death. We model mortality for people with and without diabetes as in our 2‐year analysis. Patients enter the model age 54, with 59% women, as in DiRECT. The previous analysis relied upon assumptions as to maintenance of remission beyond year 2, whereas at 5 years maintenance is mature in the sense that relatively few participants remained free of type 2 diabetes. Consequently. while economic evaluation often requires extrapolation well beyond the period of follow‐up in clinical trials, to capture relevant costs and outcomes, analysis limited to 5‐year outcomes may provide evidence of cost‐effectiveness without reliance on modelling of future outcomes. We therefore report 5 year‐based costs and QALYs within the lifetime‐based analysis.

**FIGURE 1:**
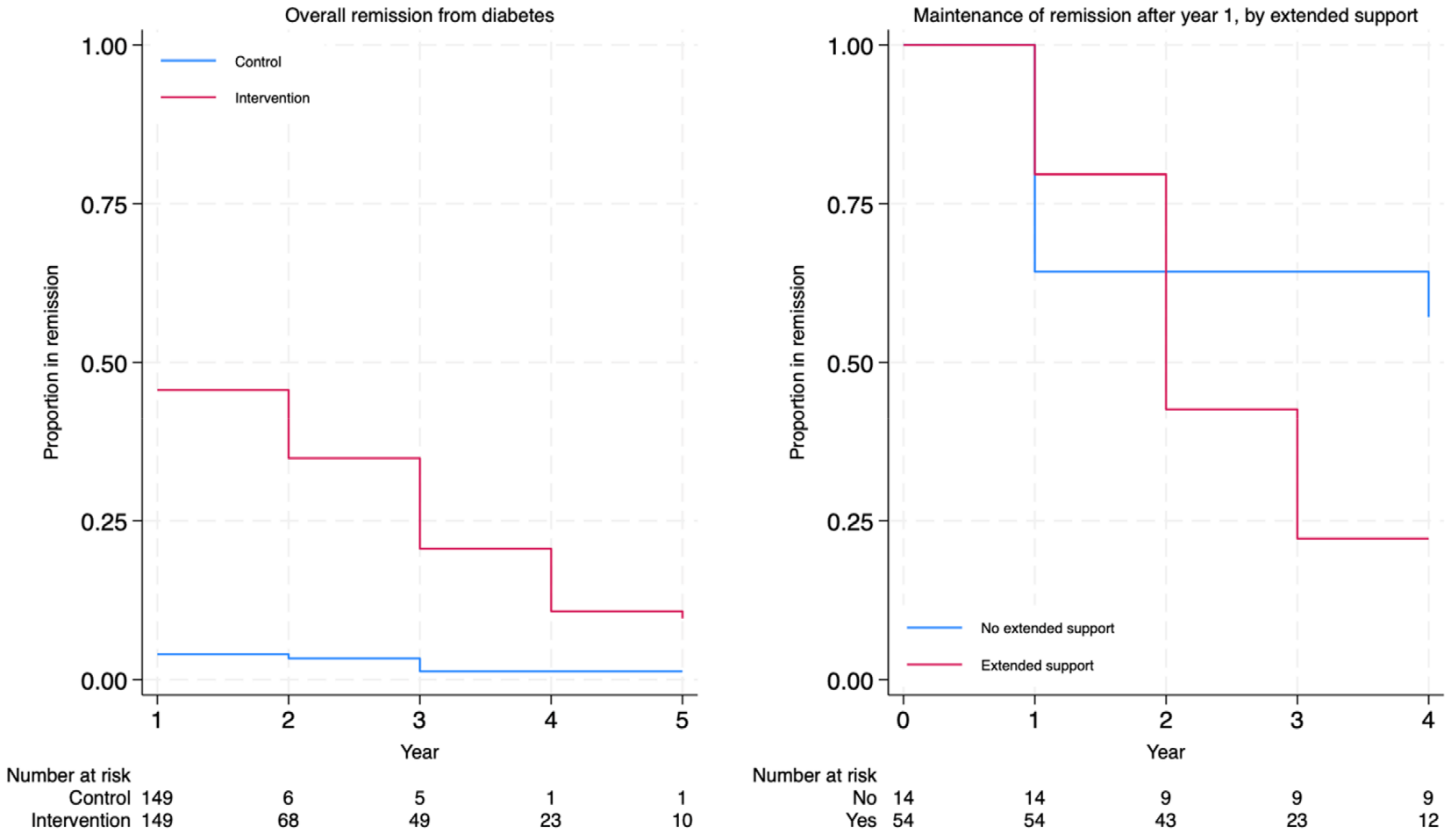
Remission from type 2 diabetes: (a) Overall remission from type 2 diabetes. (b) Maintenance of remission after year 1, by extended support.

The 5‐year data included time to relapse from remission from type 2 diabetes, intervention costs, routine healthcare resource use, including primary, secondary, and tertiary care contacts, anti‐hypertensive and anti‐diabetic medicines. Health‐related quality of life in the form of the EuroQol EQ‐5D‐3L (UK value set) was also considered. Resource use was recorded for all participants (including via GP record), irrespective of study completion or achievement of remission of diabetes. Intervention costs, healthcare costs, and time in remission were based on data recorded over the 5‐year study. A lifetime horizon was adopted, recognising the potential costs and outcomes over DiRECT participants' remaining life expectancy, including years of life saved, alongside an NHS perspective for costing. For costing of healthcare resource use, the same sources were used as far as possible* to update costs in the year 2 analysis (based on 2017/18 NHS reference costs)^9^ at the end of the extension study (NHS reference cost) presented at 2020–21 prices. As for the year 2 analysis, we applied the most recently available Drug Tariff costs.^10^ Standard UK discounting of 3.5% per annum was applied for costs and outcomes.^11^ A flow chart visually documenting participants over the course of the 5‐year study from randomisation with data available at each time point is produced in the main paper, Figure 1.^8^

As described previously, DiRECT intervention resource included initial training of practitioners, sachets of low‐energy formula diet, practitioner monitoring appointments and the tailored Counterweight‐Plus workbooks issued to each participant.^12^ Costs of the sessions included practitioners' attendance time and standard Counterweight‐Plus materials. The number of sachets issued to each participant and the number and duration of practitioner appointments were collected prospectively throughout the study. Formula diet issued over the course of the study included initial total diet replacement (8–12 weeks) followed by further low‐energy food replacement during the planned phases of food reintroduction and weight loss management, with recourse to a ‘rescue’ period of low‐energy formula diet in attempt to restore weight loss and diabetes remission. We maintain the original intervention costs as incurred during the trial. A proportion of people in the intervention arm accepted an invitation of extended support beyond the 2‐year horizon of the randomised trial. We include intervention resources consumed over this extended support period. Annual intervention costs were estimated as the average for participants remaining in the study in each year of follow‐up.

Lifetime healthcare costs, in addition to the medium‐term intervention costs, were modelled based on analysis of the 5‐year DiRECT resource use data, analysed by Kaplan–Meier sampling average,^13^ with average annual costs for participants with observations assumed to be representative of all participants modelled to remain alive. Details of all primary and secondary care visits for each of the participants were obtained directly from the participating general practitioner (GP) practice records. Unit costs were assigned to each visit, recognising the type of primary care contact or secondary care visit. Medication use was costed based on dose, frequency and start and end dates of individual participants' medication records in each participating GP practice. Hospitalisation costs were estimated by matching reason for admission and recorded length of stay in DiRECT to the appropriate NHS reference cost (including costing excess bed‐days, where length of stay recorded in DiRECT exceeded the national average using the 2020/21 National Tariff Payment System for trim points and costs by specialty).^14^ Unit costs were obtained from published national sources: staff costs from the Personal Social Service Research Unit 2020/21,^15^ NHS reference costs from English National Schedule of NHS costs 2020/21,^16^ and British National Formulary for drug tariffs.^10^ Other than for loss of follow‐up, missing resource use data were negligible, as all resource use data were obtained directly from participating GP practices. These costs were aggregated for each participant for each year of follow‐up, and average costs for those remaining under observation in each year of follow‐up calculated by Kaplan–Meier sampling average.^13^ This estimates mean costs by follow‐up year and treatment arm based on available data, which are then adopted as model inputs for the proportion of participants modelled to remain alive, under the assumption that the costs as observed for each time period are representative of the modelled surviving cohort. We assigned average healthcare costs by treatment arm and year to the modelled proportions of people surviving at each time point. For extrapolation beyond year 5, we based healthcare costs on the control arm's year‐5 cost for those not in remission and applied a cost‐saving for remission (in either arm), estimated as the average proportional cost‐saving for remission compared with non‐remission over years 2–5.

Time in remission was based on maintenance of remission after reversal of type 2 diabetes at 12 months. Though several participants achieved remission after 12 months (five intervention participants gained remission during year 2), these remissions were short lived (only one persisted beyond year 3), and may be less likely outside the context of a clinical trial with extended support, and we did not recognise these later remissions in the present analysis. For those in remission at 12 months we assumed this was maintained until the first occasion where remission was recorded as having been reversed. For annual records where remission status was not recorded, we deemed remission reversed at this point if the next available record showed this to be the case. Otherwise, participants were censored at the first occasion where no further record of remission status was available. Probabilities for remission at each annual interval were then estimated by Kaplan–Meier analysis. We made no adjustment for participants receiving continued support in the extension study but did compare the rate of loss of remission in the different groups to assess the extent to which extended support might have influenced overall remission. We assumed that the proportion remaining in remission at 5 years would continue to decline at the rate observed in the intervention arm between years 1 and 5. As in our previous analysis, we assumed that remission would not persist beyond a maximum of 10 years. We applied rates of mortality for people free of diabetes during remission and with diabetes otherwise, as in our earlier modelling.^9^, ^17^ Thus, while in remission, a lower rate of mortality was assumed than for people with diabetes, based on analysis of Scottish mortality data in people with and without diabetes.

Health‐related quality‐of‐life data (EQ‐5D‐3L) were recorded for 2 years. Beyond this point, further data were available only for participants in the intervention arm who participated in the extension study. Given the limited data available to contrast the intervention arm and control participants' utilities, we fitted a generalised estimating equation to model health‐related quality of life by remission status, rather than treatment arm. That analysis accounted for subjects' repeated measures of utility at baseline and subsequent annual visits. Inclusion of baseline characteristics in addition to time‐varying remission status was explored. In the model, we assigned all surviving participants a utility based on UK population norms and applied relative decrements for diabetes and remission based on the utility scores predicted by the regression compared with that for people in the general population at the baseline age and sex in DiRECT.

We performed analyses probabilistically using 1000 bootstrap iterations of the DiRECT data.^18^ Bootstrapping of the DiRECT data covered remission from diabetes and resource use in terms of both intervention (diet formula and related practitioner visits) and other annual healthcare resource use (primary and secondary care, medicines, and hospitalisations). Alongside each bootstrap iteration, we applied Monte Carlo simulation of utility estimates from the fitted EQ‐5D regression and beta distributions for mortality by diabetes status.

In a scenario analysis, we limit the time horizon to that of the follow‐up period in DiRECT, that is, 5 years. We also report a number of scenario analyses designed to test the impact of substantial variation in key inputs to the analysis. These analyses were designed to reduce the benefit derived from periods of remission by limiting the health benefits that subsequently accrue in terms of mortality or quality of life, including a scenario where remission over the course of DiRECT is assumed to be only half that modelled in the base case (including at 1 and 2 years). For that scenario, we apply Kaplan–Meier sampling average healthcare costs based on treatment arm *and* remission status, rather than as estimated by treatment arm only, to account for the fact cost‐savings would be expected to be lower in this eventuality; intervention costs in this scenario remain assigned based on treatment arm independent of remission status.

We also explored the impact of receipt of extended intervention support, and achievement of remission at 12 months, irrespective of its longevity, on annual healthcare costs. These analyses (using a generalised linear regression model – gamma with log link – accounting for participant level clusters) were performed to provide additional insight regarding the role of extended intervention support and remission, rather than to provide alternative parameter estimates for the cost‐effectiveness model.

Statistical analyses were performed using Stata V.18 and modelling was undertaken in Microsoft Excel.

## 3 RESULTS

Among those with available data, 10% were recorded as in remission at 5 years (13% in the extension participants).^8^ By Kaplan–Meier estimation, 9.7% of the intervention arm participants maintained remission beyond 5 years. Though 86 (intervention arm) participants entered the extension group at 2 years, the number of these in remission at this point was 54; just 14 of those in remission did not enter the extension. This limits the potential to assess the impact of extended support, however, though acceptance of extended support was associated with greater remission in DiRECT^8^ maintenance of achieved remission did not appear to be any greater among those who did receive extended support than among those who did not, and we made no adjustment for any impact extended support might have had on maintenance of remission. Mean (undiscounted) life expectancy for the control arm was modelled to be 23.5 life years. Years in remission for the intervention arm were modelled over the first 5 years as 1.42 on average. We model time in remission as having a lower risk for mortality, and this leads to an additional life expectancy of 0.08 life years per person for the intervention arm.

**FIGURE 2:**
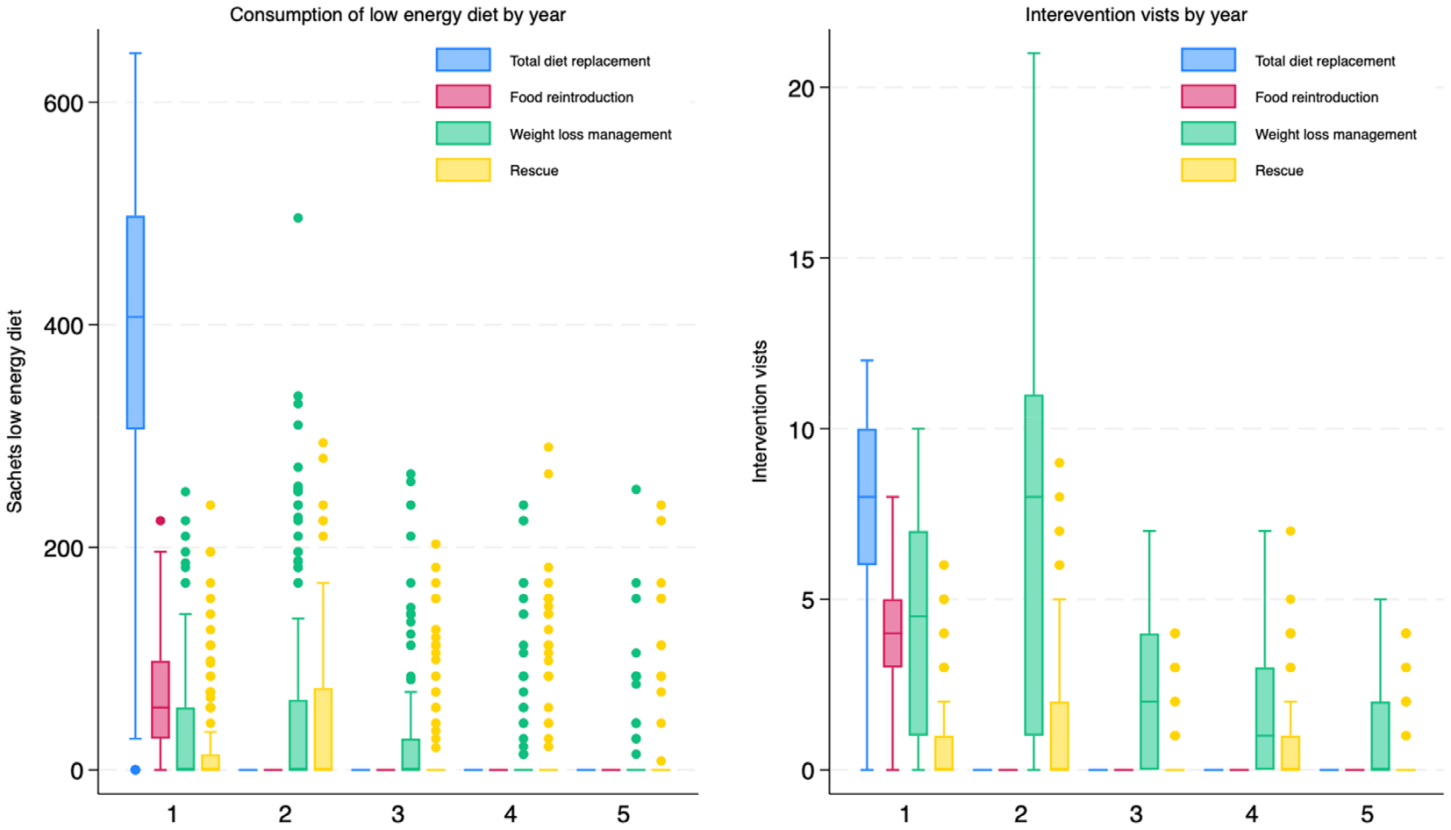
Intervention resource use: (a) Consumption of low‐energy diet by year. (b) Intervention visits by year.

We previously reported intervention resource use for years one and two of DiRECT. The initial total diet replacement phase accounted for approximately 75% of the total first year consumption, with sachets consumed during subsequent food replacement accounting for approximately a further 12% (Figure 1). As shown in Figure 2, during the first year an average of approximately 500 sachets of low‐energy formula diet were issued to each participant. In year 2 diet replacement was principally in relation to continued weight loss management. Extended support added marginally to the intervention resource consumed in subsequent years. Practitioner visits associated with delivery of the Counterweight‐Plus intervention correlated with the number of sachets issued, with the majority of visits taking place in years one and two. The total intervention (set‐up, formula diet and practice visits) cost was £1691 (95% CI £1566, £1822) per participant over 5 years (Table 1). No further intervention costs are applied beyond year 5.

**TABLE 1:** Intervention resource use.

	Intervention year				
	1	2	3	4	5
*N*	148	144	141	139	136
Low‐energy diet (sachets)					
Total diet replacement	388.4 (153.8)	Not applicable			
Food reintroduction	62.6 (49.1)	Not applicable			
Weight loss management	34.3 (53.0)	55.8 (91.9)	24.9 (53.4)	17.4 (47.1)	11.9 (36.2)
Rescue	24.4 (49.8)	40.0 (66.3)	19.4 (44.7)	26.6 (57.6)	16.1 (47.2)
Total sachets	509.8 (221.6)	95.9 (116.0)	44.3 (73.6)	44.0 (82.7)	28.1 (70.1)
Intervention visits					
Total diet replacement	7.8 (2.8)	Not applicable			
Food reintroduction	3.6 (1.8)	Not applicable			
Weight loss management	4.1 (3.0)	6.6 (5.2)	2.1 (2.1)	1.8 (2.0)	1.2 (1.4)
Rescue	0.8 (1.6)	1.3 (2.2)	0.5 (1.1)	0.7 (1.4)	0.5 (1.1)
Total visits	16.4 (6.0)	7.9 (5.6)	2.7 (2.6)	2.5 (2.9)	1.6 (2.0)

Over the study average healthcare costs per annum were ~£480 per participant lower (Table 2) in the intervention arm (*p*‐value, p.0.034). Reduced hospital admissions accounted for a major part of this saving, with the remainder being across primary and secondary care, and anti‐diabetic and anti‐hypertensive medications.

**TABLE 2:** Healthcare resource use per annum.

	Healthcare contacts p.a.		Healthcare cost (£) p.a.	
	Control	Intervention	Control	Intervention
	Mean (SD)	Mean (SD)	Mean (SD)	Mean (SD)
N (participant years)	674	708	674	708
GP	5.13 (5.44)	4.41 (4.69)		
Nurse	3.45 (3.14)	2.66 (2.43)		
Healthcare assistant	0.62 (1.25)	0.64 (1.17)		
Primary care	9.20 (6.87)	7.71 (6.20)	428 (330)	357 (294)
Other	0.59 (1.83)	0.63 (1.51)	44 (149)	50 (163)
Secondary care	1.37 (1.72)	1.14 (1.48)	347 (535)	288 (487)
Hospital	0.15 (0.52)	0.10 (0.35)	690 (3136)	412 (1792)
Medicines			300 (663)	222 (514)
Total			1809 (3577)	1329 (2211)

While Table 2 provides a summary of average annual costs, we assigned average healthcare costs by treatment arm and year to the modelled proportions of people surviving at each time point. We based healthcare costs beyond 5 years on the control arm's year‐5 cost for those not in remission and applied a cost‐saving for remission (in either arm), estimated as the average proportional cost‐saving for remission compared with non‐remission over years 2 to 5.

Total healthcare cost‐savings (excluding intervention costs) over 5 years amounted to £2091. Over the lifetime horizon, this increased marginally to £2187 (95% confidence interval £342, £4517). These cost‐savings offset the intervention costs, resulting in total incremental cost‐saving of £496 (Table 3) for the intervention arm (95% confidence interval £2858 cost‐saving, £1339 incremental cost).

**TABLE 3:** Summary of lifetime cost‐effectiveness.

	CW‐Plus	Control
Costs (£)		
Set‐up	68	0
Sachets	904 (840, 968)	0
Time	507 (473, 544)	0
Sachets	161 (118, 204)	0
Time	120 (100, 140)	0
Intervention total (5 yrs)	1691 (1566, 1822)	0 (0, 0)
Health total (5 yrs)	5837 (4823, 6927)	7928 (6383, 9683)
Healthcare incremental (5 years)		−2091.4 (−4170, −252)
Incremental (5 years)		−400 (−2491, 1419)
Healthcare total	26,288 (20,510, 33,622)	28,476 (21,676, 37,055)
Healthcare total incremental		−2187 (−4517, −342)
Total	27,980 (22,182, 35,249)	28,476 (21,676, 37,055)
Incremental total cost		−496 (−2858, 1339)
Outcomes		
Remission free years	1.46 (1.09, 2)	0.15 (0, 0.43)
Life years	23.57 (23.43, 24)	23.48 (23.33, 23.6)
QALY (5 years)	3.41 (3.33, 4)	3.37 (3.25, 3.49)
Incremental QALY (5 years)		0.04 (0.01, 0.08)
QALY total	11.02 (10.69, 11)	10.94 (10.56, 11.34)
Incremental total QALY		0.08 (0.04, 0.15)
Cost per QALY		CW‐Plus dominant

**FIGURE 3:**
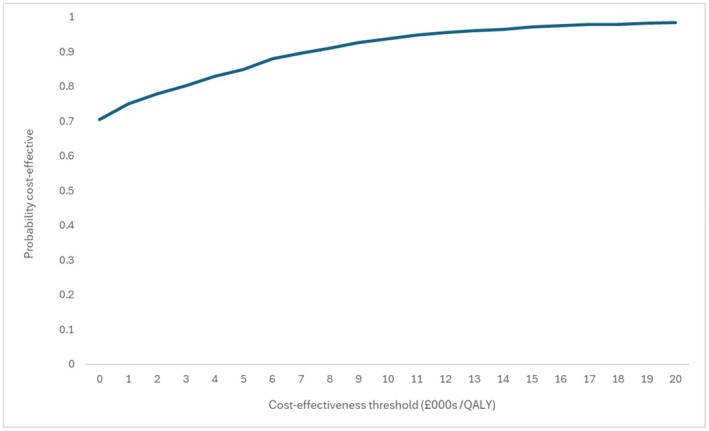
Cost‐effectiveness acceptability curve.

The utility analysis showed remission to be independently associated with an improvement in quality of life of 0.037 (*p* = 0.016). Neither age, sex, duration of diabetes, weight, nor HbA1c at baseline were significant predictors of utility, either when all were included in the model or after stepwise removal. In combination with the modelled advantage in survival, this produces an incremental QALY gain for the intervention, which therefore dominates the control arm (is both more effective and less costly). Over 5 years the modelled QALY gain was 0.043 (95% confidence interval 0.014, 0.078). Reduced mortality during remission contributes further health gain over the lifetime horizon (Table 3). The probability that the intervention arm is cost‐saving is 0.67, with the probability that intervention is cost‐effective rising to 0.98 when QALY gains are valued at a threshold of £20,000 per QALY (Figure 3).

The probability that the intervention is cost‐saving remained above 0.60 over a range of scenario analyses. Restricting the impact of remission on mortality and quality of life both reduced the modelled QALY gains. While adjusting quality‐of‐life assumptions can have no impact on costs, reducing the impact of remission on mortality increased cost‐savings, as additional healthcare costs in added years of life are reduced.

Scenario analyses (Table 4) assessed the impact of assumptions designed to limit the modelled benefits from remission. Restricting the analysis to a 5‐year period has little impact on incremental costs, as these are mostly driven by the initial intervention and remission. Though the lifetime gains in terms of life expectancy and QALYs are by definition limited by the shorter time horizon, the intervention remained dominant. Similarly, though constraints on the degree to which remission translates into health gains in terms of either mortality or quality of life reduced modelled incremental QALYs, these remained positive. In the base case, we assigned general annual healthcare costs on the basis of treatment arm. In scenario analyses, we explored assignment of these costs separately within each treatment arm based on remission status. This has negligible impact on incremental costs but supports a further analysis in which the proportion of intervention participants in remission is reduced, with a consequent increase in general healthcare costs with lesser remission. With very substantial reductions in remission in the intervention arm, incremental health gains persist. With even a 50% reduction, and with no reduction in intervention costs in this analysis relative to the base case, cost‐savings continued to be modelled for the intervention arm.

**TABLE 4:** Scenario analyses.

	Incremental cost (£)	Incremental QALY	Probability cost‐saving	Probability cost‐effective^a^
Base case	−496 (−2858, 1339)	0.08 (0.05, 0.16)	0.705	0.984
50% impact of remission on mortality	−547 (−2934, 1283)	0.06 (0.03, 0.13)	0.730	0.974
Zero impact of remission on mortality	−597 (−3021, 1239)	0.04 (0.01, 0.09)	0.749	0.938
50% impact of remission on quality of life	−496 (−2858, 1339)	0.06 (0.04, 0.12)	0.705	0.968
Zero impact of remission on quality of life	−496 (−2858, 1339)	0.04 (0.03, 0.08)	0.705	0.942
Treatment remission‐based costing	466 (−2741, 1373)	0.08 (0.05, 0.16)	0.695	0.980
5‐year time horizon	−400 (−2491, 1419)	0.04 (0.01, 0.08)	0.645	0.902
50% reduction in 12‐month remission	−83 (−2293, 1791)	0.04 (0.01, 0.08)	0.53	0.828

^a^ Cost‐effectiveness threshold of 20,000 per QALY.

We explored general healthcare costs further, though without any implication for the cost‐effectiveness analysis. Remission was associated with significantly lower average healthcare costs per annum compared with non‐remission, of ~£730 (*p* = 0.0189). When considering only the post‐12‐month period (i.e. after assessment of remission), this advantage increased to £894 (*p* = 0.012). The few people in the control arm who did achieve remission incurred substantially lower average annual costs while in remission, compared with control participants not in remission. However, even while not in remission, intervention participants (with greater weight losses) also incurred substantially lower average annual costs than control arm participants.

Though not statistically significant, in the post‐2‐year period, the average annual cost for those not in remission in the intervention arm was lower for those in the extended support group. This was also the case in the first 2 years, however, and extended support was not associated with lower costs in non‐remission (*p* = 0.55) Prior remission was also associated with lower average annual costs. In the intervention arm, remission at 12 months was associated on average with ~£650 lower annual healthcare costs (*p* = 0.015), irrespective of subsequent maintenance of remission.

## 4 DISCUSSION

The 5‐year results from DiRECT provided evidence of sustained weight loss and remission from type 2 diabetes in a small proportion of those in remission at 2 years. Cost‐effectiveness analysis based on 2‐year DiRECT data suggested the Counterweight‐Plus diet programme implemented in DiRECT would be cost‐saving within 6 years. That analysis relied on published estimates for diabetes‐related costs applied to those not in remission.^19^ The 5‐year follow‐up data for DiRECT provide actual healthcare resource use data drawn from GP electronic records. The modelled total costs in the present analysis are therefore a closer reflection of the impact that weight loss induced remission of type 2 diabetes may be expected to have. We modelled total costs based on the observed mean costs for people remaining in the study in each arm at each year of follow‐up. Though we found remission to be associated with lower healthcare costs, particularly in the period after the first 12 months during which the intervention was primarily delivered, this did not fully account for the lower costs in the intervention arm. We found that remission at 12 months was associated with reduced subsequent costs irrespective of maintenance of remission. It may be that weight loss without remission explains this, as weight is the driver of diabetes and many other linked complications.^20^ While both weight regain and loss of remission were common in DiRECT, of 32 people with loss of remission between 2 and 5 years, for whom weight data were available, ~50% saw either a further weight reduction or an increase of less than 1 kg per year.^21^ We did not model weight loss itself, but the modelled total costs will reflect this.

There was continued support over the period, both in terms of formula diet products and practitioner contacts among those in the intervention arm who accepted the invitation to participate in the 5‐year extension study. Our earlier cost‐effectiveness analysis also assumed some continued provision of the intervention for those in remission. The additional resource associated with the intervention beyond two years, however, was relatively modest, and the total intervention cost over 5 years amounted to £1691 (95% confidence interval £1566, £1822). The total healthcare resource use (excluding intervention costs) was modelled to be significantly lower for the intervention arm, more than offsetting the total cost of intervention delivery within 5 years. Annual healthcare costs, generated from electronic records, were censored in only 2% of intervention participants in remission at 2 years (three extension participants); there was no indication that censoring, which was slightly higher among both intervention participants not in remission at 2 years and non‐intervention participants, would have favoured the intervention arm. Continued support was offered to participants in DiRECT, rather than being a distinct strategy for which cost‐effectiveness can be estimated. Though this support may have contributed in part to maintenance of remission and lower resource use, we found no difference in either the maintenance of remission or general healthcare costs among those not in remission according to receipt of extended support. However, we cannot know how participants in the extended support group would have fared had this further support not been offered.

The Look AHEAD study^22^, ^23^ reported a 51% decrease in cardiovascular disease events for those who maintained remission (4 years), along with reduced chronic kidney disease. We applied mortality reductions for people in remission. Though both DiRECT and Look AHEAD have demonstrated clinical benefits from weight loss and remission that may be expected to reduce mortality, this cannot be explicitly shown for the Counterweight‐Plus energy diet intervention. Reduced mortality, even over the short‐term, can yield substantial benefits in terms of average life expectancy if subsequent loss of remission is not assumed to carry any greater hazard than would continue type 2 diabetes. Though the 5‐year DiRECT data show relatively few people still in remission, the use of a lifetime horizon allows these future benefits from avoided mortality to be taken into account. Our assumption that remission is associated with no greater mortality than in people free of diabetes in the observational study on which we base this, however, is central to our longer term modelling. Given the uncertainties around this, we considered scenarios in which the benefit of remission in terms of mortality was either reduced or completely removed. Even in the latter case, though substantially reduced, QALY gains arise due to the improvement in quality of life attributed to remission. Whereas our modelled life expectancy gains rely on mortality patterns for non‐type 2 diabetes populations applying to people in temporary remission from diabetes, the quality of life benefit from remission is based on EQ‐5D‐3L data collected in DiRECT. Though a benefit in the intervention arm was reported in DiRECT based on the visual analogue scale,^8^ that is not the basis on which QALYs are modelled here, as economic evaluation employs the preference‐based social tariff.^24^ The EQ‐5D‐3L analysis showed no direct benefit in quality of life for the intervention compared with control arm at 2 years. Even had the 5‐year data demonstrated a benefit, this would not have involved any further quality‐of‐life data for the control arm. The lack of a direct comparison of utility between treatment arms might be seen as a limitation arising from there being no EQ‐5D data after 2 years available for the control arm. Cost‐effectiveness analyses, however, often rely on quality‐of‐life estimates for health states (the occupancy of which is modelled to differ between arms), rather than directly comparable estimates between trial arms. While the contribution of mortality to total QALY gains relies on external evidence that is assumed applicable to the present context, there is evidence from DiRECT to support there being QALY gains from the Counterweight‐Plus intervention, based on the effect remission was seen to have on EQ‐5D‐3L within the intervention arm. A limitation concerning our estimation of the effect of remission on utility is its reliance on follow‐up data in DiRECT in the self‐selected group of extension study participants, though the majority of those in remission did participate in the extension study. Assuming a diminished impact of remission on quality of life naturally reduces the QALY gain associated with the intervention, though these remain positive.

As in our earlier analysis, we focused on remission of diabetes, rather than weight loss, and did not attempt to model any marginal health impacts of improved weight management among those not in remission of diabetes. Though our approach will have captured the effects of this in terms of lower healthcare costs, a limitation of our approach is the exclusion of any health benefits over and above remission. Given our focus on remission, we did not seek to include a time‐varying effect of weight in our utility analysis. Applying any such effect would have required a more complex model design and departed from our 2‐year analysis.

Analysis of Scottish Care Information (SCI) Diabetes registry data has reported mean annual cost per person (including productivity) without cardiovascular disease and not at high risk of £2500. Our annual estimates for non‐intervention participants ranged between £1500 and £2500 per year, though exclusive of productivity costs are of similar order. We based our current analysis on costs as observed in DiRECT, whereas our earlier analysis modelled increasing cost pressures with greater duration of diabetes, and the SCI analysis reported increasing costs following cardiovascular events. Had we maintained a correspondence between remission and delayed future healthcare costs, our modelled savings could be expected to have been greater.

The analysis of EQ‐5D employed all observations of remission, whenever these were recorded. In assigning remission in the model, however, we treated a number of participants who achieved remission after 12 months as being non‐remission and did not account for any subsequent remission among participants who had at any stage after 12 months lost their remission. Though a simplification of the pattern of remission seen in DiRECT, these late remissions seen in DiRECT might be less likely in a routine care setting.

Our analysis reflects the professional support from nurses and dietitians in DiRECT and the extension support to 5 years which was mainly face‐to‐face contact, with some remote support during the COVID restrictions. Most weight management programmes for remission of type 2 diabetes, including Counterweight, are now provided entirely remotely, with substantially lower programme delivery costs and comparable results. Participants in the NHS ‘Path to Remission’ programme,^25^, ^26^ which provides a DiRECT‐like intervention (based on DiRECT), have the option to receive this support from a coach in‐person or digitally. Reported remission rates among participants completing 12 months with two available HbA1c recordings have been lower than were seen in DiRECT, ~32%. In our scenario analysis, assuming only half the initial remission seen in DiRECT, the intervention remained cost‐effective, though with a lower probability of being cost‐saving than in our base case, despite this analysis reflecting in‐person face‐to‐face programme delivery costs. Research is now turning to evaluation of eHealth‐based programmes as an alternative to face‐to‐face.^27^

Much of the cost‐saving modelled here can be attributed to reduced hospitalisations in the intervention arm. Anti‐diabetes and hypertensive medications were also less costly. There was some use of weight loss medication during the DiRECT trial and follow‐up, though this can be expected to continue to grow. Interventions such as Counterweight‐Plus may provide a less costly alternative strategy for weight loss and remission of type 2 diabetes. This point may be particularly relevant to low‐ and middle‐income countries, where weight loss medicines will simply be unaffordable for many years to come.

## CONFLICT OF INTEREST STATEMENT

MEJL reports grants and personal fees unconnected with the present work from Counterweight, Novo Nordisk, Novartis, and Eli Lilly. RT has received lecture fees from Novartis and Eli Lilly and has served on an advisory panel for Wilmington Healthcare. NS reports grants and personal fees from AstraZeneca, Boehringer Ingelheim, Novartis, and Roche Diagnostics and personal fees from Abbott Laboratories, Amgen, Eli Lilly, Hanmi Pharmaceuticals, Janssen, Merck Sharp & Dohme, Novo Nordisk, Pfizer, and Sanofi outside the submitted work. WSL reports support for conference attendance from Cambridge Weight Plan outside the submitted work. LM reports employment by Counterweight during the conduct of study, was previously a shareholder in Counterweight and previous employment from Cambridge Weight Plan outside the submitted work. All other authors declare no competing interests.

## Footnotes

* There were some differences in level of disaggregation given high level summaries only were produced during COVID‐19.
